# The effects of the Covid-19 pandemic on the stress and depression levels of new mothers

**DOI:** 10.1093/eurpub/ckac129.747

**Published:** 2022-10-25

**Authors:** C Florea, J Preiß, M Angerer, M Schabus

**Affiliations:** Psychology, Paris-Lodron University of Salzburg, Salzburg, Austria

## Abstract

**Background:**

New mothers are a particularly vulnerable group during the COVID-19 pandemic, due both to the higher depression and stress levels associated with early child care and to the risk of a COVID-19 infection. We investigated the effects of the pandemic on the childbirth experience, stress and depression levels in this population.

**Methods:**

This cross-sectional, explorative study included 1964 Austrian and German mothers who gave birth between 16.03.2020 and 01.07.2021 and completed an online survey between 18.05.2021 and 01.07.2021. This contained the Childbirth Experience Questionnaire (CEQ2), the Edinburgh Postnatal Depression Scale (EPDS), and the Perceived Stress Scale (PSS), as well as three custom-made scales: a birth risk score (risk factors for a poor birth outcome), a pandemic repercussions score (perceived effects on different aspects of personal life), and a social support score (how emotionally supported they feel). We computed post-hoc multilinear regression models to evaluate which factors can predict the CEQ2, PSS and EPDS scores.

**Results:**

Mothers had a worse birth experience, perceived less stress and had more depressive symptoms during the pandemic than previously reported cohorts. The CEQ2 was predicted by the birth risk (negatively), the access to a midwife (positively) and the perception of sufficient access of the visitors in the hospital (positively) (adjusted R2 = 0.26, F(4, 1738) = 155.64, p < 0.001). The PSS was predicted by the pandemic repercussions (positively), the social support (negatively), and the presence of a coping mechanism (negatively) (adjusted R2 = 0.28, F(4, 1959) = 195.1, p < 0.001). The EPDS was similarly predicted by the same factors as the PSS (adjusted R2 = 0.28, F(4, 1959) = 189.59, p < 0.001).

**Conclusions:**

Social support and strong coping mechanisms can lower the stress and depression scores. Instructing the population about how to improve these factors might be a target for future social policies.

**Key messages:**

• Compared to historical cohorts, mothers who gave birth during the pandemic had a worse birth experience, and, postnatally, perceived less stress but had more depressive symptoms.

• The visitors’ access to the hospital and the mother’s access to a midwife impacted the birth experience, while the social support and the coping mechanisms affected the stress and depression scores.

